# Psychoactive Pharmaceuticals Induce Fish Gene Expression Profiles Associated with Human Idiopathic Autism

**DOI:** 10.1371/journal.pone.0032917

**Published:** 2012-06-06

**Authors:** Michael A. Thomas, Rebecca D. Klaper

**Affiliations:** 1 Department of Biological Sciences, Idaho State University School, Pocatello, Idaho, United States of America; 2 School of Freshwater Sciences, University of Wisconsin-Milwaukee, Milwaukee, Wisconsin, United States of America; Alexander Flemming Biomedical Sciences Research Center, Greece

## Abstract

Idiopathic autism, caused by genetic susceptibility interacting with unknown environmental triggers, has increased dramatically in the past 25 years. Identifying environmental triggers has been difficult due to poorly understood pathophysiology and subjective definitions of autism. The use of antidepressants by pregnant women has been associated with autism. These and other unmetabolized psychoactive pharmaceuticals (UPPs) have also been found in drinking water from surface sources, providing another possible exposure route and raising questions about human health consequences. Here, we examined gene expression patterns of fathead minnows treated with a mixture of three psychoactive pharmaceuticals (fluoxetine, venlafaxine & carbamazepine) in dosages intended to be similar to the highest observed conservative estimates of environmental concentrations. We conducted microarray experiments examining brain tissue of fish exposed to individual pharmaceuticals and a mixture of all three. We used gene-class analysis to test for enrichment of gene sets involved with ten human neurological disorders. Only sets associated with idiopathic autism were unambiguously enriched. We found that UPPs induce autism-like gene expression patterns in fish. Our findings suggest a new potential trigger for idiopathic autism in genetically susceptible individuals involving an overlooked source of environmental contamination.

## Introduction

Autism spectrum disorders (ASD) are characterized by stereotyped behaviors and impaired social skills, typically diagnosed by three years of age [1]–[3]. Idiopathic ASD, caused by genetic susceptibility factors [4]–[6] interacting with unknown environmental triggers [7], [8], has increased dramatically in the past 25 years [9], [10]. Identifying environmental triggers has been difficult due to the poorly understood pathophysiology of ASD and broad, subjective case definitions.

In order to serve as such a trigger, a candidate teratogen must have a biologically plausible etiological mechanism, exist in sufficient environmental concentrations, be capable of passing from mother to fetus and across the fetal blood-brain barrier (if one assumes prenatal exposure), and have experienced historical increases in environmental concentration parallel with observed increases in ASD prevalence.

Coincident with the observed increase in ASD prevalence is the introduction of modern rationally designed psychoactive pharmaceuticals, beginning with selective serotonin re-uptake inhibitors (SSRIs) in 1987 (initially fluoxetine, FLX; now 9+ versions), and serotonin–norepinephrine reuptake inhibitors (SNRIs) in 1994 (initially venlafaxine, VNX; now 8+ versions). These pharmaceuticals result in an increase in the neurotransmitter serotonin, which is responsible for the regulation of neural activity and other physiological functions [11]. The leaky blood brain barrier of the fetus and infants is permeable to many compounds, making this population particularly vulnerable to the effects of serotonin. Maternal exposure to SSRIs result in elevated fetal plasma serotonin levels, which has been associated with autism [12]. In other work, rat pups exposed prenatally to SSRIs exhibited behaviors associated with ASD [13].

The use of SSRIs and SNRIs by pregnant women to treat depression and other psychological disorders has been associated with low APGAR (Appearance, Pulse, Grimace, Activity, Respiration) scores [14], increased risk of spontaneous abortion [15], several other consequences for children [16]–[20], and, recently, ASD [21]. Several other classes of psychoactive pharmaceuticals are also known autism risk factors when taken prenatally [22], [23], including valproic acid used to induce the autism rat model [24], [25]. Since the 1960s, for example, pregnant women have used carbamazepine (CBZ) for control of seizures, despite its potential association with developmental issues [26]. Studies of these drugs considered only effects of maternal usage of clinical dosages [21], [27]; while widespread, clinical usage of antidepressants is insufficient to account for recent increases in autism prevalence.

There is an alternative source of exposure to antidepressants: Unmetabolized psychoactive pharmaceuticals (UPPs) found in raw sewage, effluent from sewer treatment facilities, rivers downstream of such facilities, and, ultimately, drinking water [28], [29]. Because concentrations are so minute (typically ng to µg·L^-1^), human health consequences of UPPs remain controversial. While the highest observed concentrations of UPPs have biological effects in fish [30], these concentrations are many orders of magnitude below human clinical dosages (Table 1). While fetuses and infants may have been exposed to UPPs through maternal water consumption, it has been assumed that UPPs have no measurable and enduring effects on human health [31], [32]. However, multiple related formulations and active metabolites of UPPs present in the environment exist in complex mixtures [33], [34] that together constitute much higher dosages, especially in contamination hot-spots [35]. Therefore, we describe the experimental concentrations used here as similar to the highest observed environmental concentrations, despite the fact that experimental concentrations are an order of magnitude higher than the most recent (and probably conservative) estimates of environmental concentrations (Table 1).

**Table 1:** Observed values of psychoactive pharmaceuticals in various systems.

Source	FLX	VNX	CBZ
Experimental	10 µg·L^−1^	50 µg·L^−1^	100 µg·L^−1^
Raw sewage	0.073 µg·L^−1^ [50]	2.19 µg·L^−1^ [51]	6.3 µg·L^−1^ [52]
Wastewater treatment plant (WWTP)	0.509 µg·L^−1^ [53]	1.115 µg·L^−1^ [47]	17.3–22.0 µg·L^−1^ [54], [55]
Effluent from WWTP	0.841 µg·L^−1^ [56]	No information	1.16 µg·L^−1^ [57]
Downstream from WWTP	0.93 µg·L^−1^ [55]	0.387 µg·L^−1^ [57]	2.3 µg·L^−1^ [47]
River system	0.12 µg·L^−1^ [58]	1.31 µg·L^−1^ [51]	1.283 µg·L^−1^ [52]
Drinking water	0.014 µg·L^−1^ [59], [60]	No information	0.25 µg·L^−1^ [60], [61]

FLX, VNX and CBZ are fluoxetine, venlafaxine and carbamazepine, respectively. Values reported indicate the highest observed concentrations from various systems. Experimental treatment dosage was selected to reflect combined dosages of multiple active metabolites for each pharmaceutical.

Here, we describe results of an experiment that explores a potential association between UPPs and idiopathic ASD. We tested whether chronic exposure to a mixture of UPPs induced autism-like gene expression profiles in a model organism using gene-class analysis. We used treatments involving a mixture of UPPs similar to that observed in aquatic systems and examined expression of genes expressed by individuals with various forms of idiopathic ASD. For comparison, we examined sets of genes expressed by individuals with other neurological disorders.

### Gene Expression Analysis

We exposed fathead minnows, *Pimephales promelas*, to FLX, VNX, and CBZ in a 3-component mixture. FLX, VNX, and CBZ were chosen because they represent modern pharmaceutical classes that are highly prescribed and are among the UPPs with the highest observed environmental concentrations (Table 1). We conducted gene-class analysis of expression patterns induced by the pharmaceutical treatments using Gene Set Enrichment Analysis (GSEA) [36] and an enhanced annotation of the fathead microarray platform [37]. The gene-class analysis approach tests if a set of genes, described *a priori*, is enriched by a given treatment. The data for the present study were derived from a previous analysis of this system [38]. In that study, we found enrichment of gene sets associated with neurological development, growth and regulation by the mixture of UPPs. These genes sets were not enriched by treatments of the pharmaceuticals administered separately, and were associated with the formation and regulation of neural circuits, which may indicate formation of altered and imprecise synaptic connections and presage a failure to form typical mature neural circuits.

In the present study, we first tested the prediction that UPPs would induce fish gene expression profiles that mimic human expression profiles observed in individuals diagnosed with various neurological disorders (“ND” sets, described in Table 2). We tested 12 sets of genes associated with idiopathic ASD (broadly defined), autism secondary to known genetic defects (involving fragile X and Rett syndromes), Alzheimer’s disease, Parkinson’s disease, schizophrenia, multiple sclerosis, major depression, bipolar disorder and ADHD.

**Table 2:** “ND”: Sets of genes associated with various human neurological disorders.

Set name	Description	Number of genes (set)	Number of genes (FH microarray)	GEO link	Reference
ASD_Idiopathic	Combination of Chakrabarti, Hu & ASD_2Class; duplicates were removed (see Table S3 for details).	–	324		
ADHD_up	A comparison of molecular alterations in environmental and genetic rat models of ADHD (up-regulated genes)	50	30	GSE12457	[62]
ADHD_down	A comparison of molecular alterations in environmental and genetic rat models of ADHD (down-regulated genes)	34	20	GSE12457	[62]
Rett	Genes found to be up-regulated in females with Rett Syndrome	39	25		[63]
ASD_Secondary	Gene expression profiles of lymphoblastoid cells from individuals with fragile X syndrome and dup(15q).	67	39	GSE7329	[64]
Schizophrenia	Proteins consistently differentially expressed in the brains of SCZ patients.	30	23		[65]
Alzheimers	Up-regulated in correlation with incipient Alzheimer’s Disease, in the CA1 region of the hippocampus	345	237	GSE1297	[66]
Parkinsons	Genes associated with Parkinson’s Disease.	162	94		KEGG HSA05012
Depression	Genes upregulated in major depressive disorder (p<0.05, fold change >1.4, mean average difference >150 in at least one of the groups, called present in greater than 20% of all samples)	45	23	GSE12654	[67]
Bipolar	Genes found to be up-regulated in individuals with bipolar disorder.	71	41		[68]
MS_Bomprezzi	In an attempt to identify molecular markers indicative of disease status rather than susceptibility genes for MS, the authors show that gene expression profiling of peripheral blood mononuclear cells by cDNA microarrays can distinguish MS patients from healthy controls.	45	28		[69]
MS_Gilli	Results showed an altered expression of 347 transcripts in non-pregnant MS patients with respect to non-pregnant healthy controls.	348	216	GSE17393	[70]

Second, we tested the prediction that UPPs would induce fish gene expression profiles that mimic human expression profiles observed in individuals diagnosed with autism of various degrees of severity. The idiopathic autism set used above (described in Table 2) was derived from several independent gene expression studies with minimal overlap of gene constituents, each of which identified genes enriched in individuals diagnosed with idiopathic autism. One of these sets [39] was deconstructed into specific populations classified by severity of autism symptoms. We created a second collection consisting of 10 autism sets (“ASD” sets; Table 3): nine sets associated with gene expression in individuals diagnosed with some form of autism plus a set of susceptibility genes not associated with any known increase in gene expression but, rather, known to have either mutations (e.g., single nucleotide polymorphisms) or structural variations (e.g., copy number variation) associated with some form of autism [4].

**Table 3:** “ASD”: Collection of sets of genes associated with idiopathic autism.

Set name	Description	Number of genes (set)	Number of genes (FH microarray)	GEO link	Reference
Pinto	Genes associated with genetic susceptibility to ASD but not known to be up- or down-regulated in the disorder.	104	63		[4]
Chakrabarti	Genes related to sex steroids, neural growth, and social-emotional behavior associated with autistic traits, empathy, and Asperger’s syndrome. Included only mild cases (no severe language impairment).	66	43		[71]
Hu	Gene identified by comparisons of neurotypical vs. ASD individuals with severe language impairment (with individuals having specific genetic and chromosomal abnormalities and co-morbid disorders excluded from study).	34	22		[72]
ASD_2Class	Significantly differentially expressed genes from a 2-class SAM analysis of data from combined autistic samples and neurotypical controls, with FDR <5%	370	240	GSE15402	[39]
ASD_Mild	Significantly differentially expressed genes from a 2-class SAM analysis of data from the group with mild ASD (M) and neurotypical controls (C ), with FDR <5%	360	241	GSE15402	[39]
ASD_Severe	Significantly differentially expressed genes from a 2-class SAM analysis of data from the group with severe language impairment (L) and neurotypical controls (C ), with FDR <0.0001%	191	121	GSE15402	[39]
ASD_Shared	Common genes to all GSE15402 sets.	70	48	GSE15402	[39]
ASD_Savant	Significantly differentially expressed genes from a 2-class SAM analysis of data from the group with savant skills (S) and neurotypical controls (C ), with FDR <5%	81	60	GSE15402	[39]
Voineagu_Down	Gene down-regulated in autistic cortex; found to be enriched for gene ontology categories associated with synaptic function	209	121		[40]
Voineagu_Up	Gene up-regulated in autistic cortex; found to be enriched for gene ontology categories associated with immune and inflammatory response functions	235	132		[40]

## Results

In the analysis of the ND sets, seven of 12 sets were up-regulated, with significant enrichment of the set associated with idiopathic autism (Table 4). We also observed enrichment of two disorders with adult onset: a set associated with Parkinson’s and one set associated with MS (but not a second, independent MS set). There was no overlap between the autism set and the Parkinson’s & MS sets; 4 genes occurred in both MS and Parkinson’s sets. No other set associated with human neurological disorders was enriched, including secondary autism sets.

**Table 4:** Sets associated with human neurological disorders.

Set	Size	NES	p-value	FDR q-value
AUTISM_IDIOPATHIC	324	1.621	0.000	0.064
PARKINSONS	94	1.560	0.007	0.055
MS_GILLI	216	1.375	0.011	0.137
SCHIZOPHRENIA	23	1.232	0.181	0.364
MS_BOMPREZZI	28	1.199	0.201	0.326
ADHD_UP	30	1.187	0.222	0.275
DEPRESSION	23	1.137	0.307	0.293
ADHD_DOWN	20	–0.684	0.894	0.924
RETT	25	–0.784	0.798	1.000
ALZHEIMERS	237	–0.967	0.549	0.859
ASD_SECONDARY	39	–1.083	0.332	0.764
BIPOLAR	41	–1.172	0.217	1.000

Sets are described in Table 2; size refers to the number of genes in the set; NES is the normalized enrichment scores for the set; p-value is the nominal p-value associated with the NES; FDR q-value is the false discovery rate ratio.

In the analysis of ASD sets, all 10 sets were up-regulated, with significant enrichment of five of the nine expression-based sets (Table 5). Very few genes are shared among enriched sets. The susceptibility set was not enriched; this is significant because one would not necessarily expect genes that underlie susceptibility to also experience differential expression. Interestingly, one of the strongly enriched sets (Voineagu_Down) was identified by the study authors as genes *down-*regulated in individuals with autism [40], while its complementary set (Voineagu_Up) was not enriched. The former set was endowed with an overrepresentation of gene ontology categories associated with synapse function, while the latter had an overrepresentation of categories associated with immune and inflammatory response.

**Table 5:** Analysis of sets associated with human autism.

Set	Size	NES	p-value	FDR q-value
ASD_MILD	241	1.537	0.0000	0.1261
ASD_2CLASS	240	1.519	0.0000	0.0742
VOINEAGU_DOWN	121	1.514	0.0017	0.0511
ASD_SAVANT	60	1.474	0.0298	0.0537
ASD_SHARED	48	1.459	0.0391	0.0489
CHAKRABARTI	43	1.358	0.0769	0.0864
HU	22	1.352	0.1168	0.0777
ASD_SEVERE	121	1.261	0.0781	0.1233
VOINEAGU_UP	132	1.117	0.2092	0.2749
PINTO	63	1.050	0.3558	0.3558

Column labeled as in Table 4. Sets are described in Table 3.

Normalized enrichment scores (NES; Tables 4, 5) for the examined sets ranged from -1.172 to 1.621 for ND sets (58% up-regulated) and 1.050 to 1.537 for ASD sets (100% up-regulated). While the nominal p-value indicates significance of an NES, that value is somewhat difficult to interpret. For comparison, we analyzed 242 curated gene sets from the Molecular Signatures Database [36] corresponding to each human cytogenetic band that has at least one gene. In that analysis, 52% of the sets were up-regulated, and NES values ranged from -1.70 to 1.73. Only 5% of scores exceeded an NES of 1.40, and no set passed the false discovery rate (FDR) threshold of 0.25.

The analyses in this report consider mainly the MIX treatment of UPPs. For comparison, we included GSEA analysis results from treatments consisting of the three pharmaceuticals considered individually. As one might expect, those treatments had lower NES values and fewer enriched sets relative to the MIX treatment (Tables 6, 7), although the single-drug NES values involving Parkinson’s were all higher than the MIX treatment NES values.

**Table 6:** Single drug treatments & ASD sets.

	FLX	FLX	FLX	VNX	VNX	VNX	CBZ	CBZ	CBZ
Set	NES	p-value	FDR q-value	NES	p-value	FDR q-value	NES	p-value	FDR q-value
ASD_SAVANT	1.443	0.036	0.144	1.344	0.075	0.174	1.287	0.103	0.138
ASD_MILD	1.429	0.003	0.081	1.370	0.018	0.215	1.531	0.000	0.099
ASD_SHARED	1.362	0.066	0.089	1.331	0.088	0.143	1.115	0.244	0.322
ASD_2CLASS	1.290	0.022	0.118	1.411	0.005	0.289	1.490	0.002	0.076
ASD_SEVERE	1.199	0.115	0.172	0.979	0.514	0.634	1.342	0.039	0.155
CHAKRABARTI	1.144	0.246	0.206	0.887	0.662	0.695	1.017	0.432	0.475
VOINEAGU_DOWN	−0.837	0.831	0.809	1.116	0.220	0.443	1.288	0.054	0.172
VOINEAGU_UP	−0.899	0.701	0.906	−0.909	0.716	0.670	-1.018	0.404	0.415
PINTO	−1.061	0.339	0.677	1.018	0.420	0.627	-1.065	0.337	0.659
HU	−1.378	0.080	0.150	0.901	0.582	0.747	0.893	0.591	0.697

FLX, VNX and CBZ are fluoxetine, venlafaxine and carbamazepine, respectively. Column labeled as in Table 4. Sets are described in Table 3.

**Table 7:** Single drug treatments & ND sets.

	FLX	FLX	FLX	VNX	VNX	VNX	CBZ	CBZ	CBZ
Set	NES	p-value	FDR q-value	NES	p-value	FDR q-value	NES	p-value	FDR q-value
PARKINSONS	1.650	0.000	0.058	1.780	0.000	0.011	2.110	0.000	0.000
AUTISM_IDIOPATHIC	1.505	0.000	0.219	1.412	0.004	0.390	1.5104	0.0000	0.1930
RETT	1.164	0.237	0.738	1.079	0.338	0.452	0.7529	0.8097	0.8795
SCHIZOPHRENIA	1.104	0.316	0.661	1.164	0.251	0.497	1.2040	0.2079	0.2905
ADHD_UP	1.102	0.327	0.501	1.155	0.243	0.390	1.5102	0.0397	0.0965
MS_GILLI	0.997	0.500	0.639	1.364	0.017	0.265	1.0616	0.2827	0.4790
MS_BOMPREZZI	0.904	0.589	0.755	0.857	0.687	0.732	0.9935	0.4295	0.5343
ASD_SECONDARY	0.631	0.974	0.965	–0.749	0.896	0.870	–0.622	0.970	0.968
BIPOLAR	–0.774	0.832	0.856	–1.401	0.047	0.300	–0.812	0.773	1.000
ALZHEIMERS	–1.118	0.204	0.447	–0.847	0.906	0.928	–0.934	0.640	0.969
DEPRESSION	–1.170	0.248	0.523	0.919	0.583	0.717	1.2313	0.1871	0.3349
ADHD_DOWN	–1.715	0.004	0.019	–1.375	0.110	0.177	–1.322	0.152	0.465

FLX, VNX and CBZ are fluoxetine, venlafaxine and carbamazepine, respectively. Column labeled as in Table 4. Sets are described in Table 2.

## Discussion

We found enrichment of gene sets associated with idiopathic ASD but not of sets involving autism diagnoses secondary to other disorders (Rett and fragile X syndromes) known to be caused by specific mutations. This is significant, because it indicates that enrichment in our treatments involve only idiopathic forms of ASD.

There was no enrichment of other neurological disorders except MS (in one of two sets) and Parkinson’s. This is significant because it indicates that enrichment of the idiopathic ASD set was not simply associated with general neurological processes, pathways or systems generally common to neurological disorders. The MS set has a low NES (<1.40), and a second MS set (see Table 4) is not enriched; therefore, we are not exceedingly confident in describing that set as enriched. The other enriched non-ASD set, Parkinson’s, is more interesting, with a convincing NES and an intriguing potential connection to ASD involving similar phenomenology involving brain dysfunction [41].

A number of the genes contributing to enrichment of ASD gene sets have been implicated in other recent studies not included in our analysis. For example, Suda et al. [42] found that relative expression levels of EFNB3, PLXNA4 and ROBO2 were significantly different in individuals with autism than in neurotypical individuals; protein levels of PLXNA4 and ROBO2, but not for EFNB3, were significantly reduced in brains of individuals with autism compared to control brains. In the present study, we found 3 plexin genes (PLXNB1, PLXND1 & PLXNA3; PLXNA4 was not on the array) and ROBO2 to be strongly down-regulated in response to the MIX treatment, while EFNB3 and five related genes (EFNB1, EFNA1, EFNA2, EFNA3 & EFNA5) were up-regulated. These and other genes contributing to gene set enrichment are associated with the formation of synapses, perturbation of which may indicate an altered and imprecise synaptic connections or a failure to form mature neural circuits.

The results presented here are consistent with several recent lines of inquiry: First, the hypothesis that hyperserotonemia plays a role in autism, affecting the developing fetus and potentially involving SSRIs [27]. In that work, Hadjikhani explored a potential role of elevated serotonin levels perturbing brain development during pregnancy (in which he assumes that maternal serotonin ultimately passes the fetal blood brain barrier). The author speculated that elevated levels could be increased by maternal use of serotonin elevating pharmaceuticals (like SSRIs) or consumption of serotonin-rich foods. In the present study, serotonin levels were not measured. However, all six serotonin receptor genes on the array (HTR1A, HTR1B, HTR2C, HTR4, HTR7& SLC6A4) were strongly down-regulated in response to the MIX treatment. If this implies a consequential elevation of serotonin levels, our results would seem to be consistent with the Hadjikhani hypothesis [27] and with other recent experimental work using model organisms [13].

Second, recent evidence supports an association of antidepressants, including SSRIs, with autism [21]. In that study, Croen and colleagues found a 2-fold increase in ASD risk associated with SSRIs, with the strongest effect occurring in the first trimester. The results of the present study are consistent with this finding. However, maternal SSRI use is not sufficient to explain the increase in prevalence of ASD.

Third, there is evidence for an unambiguous environmental component involved in the etiology of autism [7]. In that study, Hallmayer and colleagues provide robust evidence that, while having a moderate genetic component, ASD also clearly involves an environmental trigger. The results of the present study are consistent with this finding, as is the assumption that the environmental trigger acts in concert with genetic susceptibility.

Fourth, there is evidence of demographic changes that may have increased the proportion of genetically susceptible individuals in contemporary populations [43]. In that study, Baron-Cohen proposed that assortative mating among genetically susceptible individuals has increased the proportion of susceptible individuals in human populations since the 1970s. Especially when coupled with increased levels of an environmental trigger, this would create circumstances in which one would expect an increase in ASD prevalence. Given that SSRIs were introduced in the mid-1980s and SNRIs in the mid-1990s, coincident with increases in ASD prevalence [10], the assortative mating hypothesis provides a framework for understanding why such a trigger is able to induce such a large effect. The results of the present study provide a potential source of exposure to psychoactive pharmaceuticals that does not involve maternal clinical usage of SSRIs.

Given the conserved nature (i.e., sequence and function) of the genes involved in the observed expression profiles, and given that the genes on the Fathead array are homologous to highly conserved human genes, it is reasonable to expect induction of humans gene expression profiles similar to the Fathead profiles. This sort of approach has been effectively used for other models of human disorders [44] and in previous investigations involving the Fathead microarray platform [37]. Here, many of the enriched sets involve genes associated with neuronal development and growth [38], which is consistent with systems and pathways known to be perturbed in the developing brain of individuals with autism [40], [45].

The concentrations used in this study were higher than observed environmental concentrations in order to account for conservative concentration estimates and the presence of related formulations and active metabolites [29], [34], [46], [47]. Future work needs to be conducted to measure the concentrations of all UPP constituents present in aquatic systems and drinking water (with appropriate temporal and geographic sampling) in order to accurately assess human exposure and health consequences.

### Conclusions

These results provide a new perspective on the etiology of idiopathic ASD and suggest new directions for research into autism’s environmental “exposome” [48]. The results of the gene expression study indicate that a mixture of UPPs can induce an ASD-like gene expression profile in a model organism. Using a low-cost model system like fathead minnow, researchers can rapidly screen potential teratogens for their ability to induce ASD-like gene expression patterns in developing brains. In order to clearly determine if UPPs are associated with idiopathic ASD in humans, future work needs to examine a wider palette of UPPs (and other potential teratogens) and results need to be validated by demonstrating treatment response in another model systems. This could involve using a mouse model, with which one could measure fetal brain expression patterns, UPP concentration in fetal blood, and concentrations of fetal neurohypophyseal hormones, following maternal treatment. Further, epidemiological studies at the individual patient level should be conducted to confirm and specify the relationship between environmental contaminants and ASDs. The mimicry of ASD-like gene expression profiles in fish, described above, does not conclusively indicate UPP induction of ASD in humans. It does, however, serve as the basis for new hypotheses regarding the etiology of idiopathic ASD.

## Materials and Methods

### Ethics Statement

All fish handling and treatments were performed at the Great Lakes WATER Institute (School of Freshwater Sciences, University of Wisconsin-Milwaukee, Milwaukee, Wisconsin) using appropriate UWM Institutional Animal Care and Use Committee (IACUC) approved protocols (approval number 0708#14).

### Fish Treatments

Full details of the fish treatments are described in a previous report [38]. Briefly, three 2-gallon tanks were used for each pharmaceutical treatment along with three tanks for a mixture treatment (containing all three pharmaceuticals in the concentrations listed in Table 1) and three tanks for control (containing no pharmaceuticals). Each tank housed five juvenile fathead minnows. Dosages of pharmaceuticals were re-administered with each change of the tank water (every 2 days). Fish were exposed to treatments for eighteen days.

### Gene Set Enrichment Analysis

Fish mRNA was pooled within a tank for microarray work (for 3 replicates per treatment) but not for qPCR validation (for 15 replicates per treatment). Details of microarray experiments, including validation by qPCR analysis of 9 genes with high rank correlation and all data files, are described in a previous report [38]. Microarray experiments conformed to MIAME guidelines and results were deposited in GEO (GSE22261).

The previous study [38] also described an altered phenotype associated with pharmaceutical treatment that involved measuring fish behavior in response to a startle stimulus modeled after predator avoidance behavior used elsewhere [49]. We found that fish behavior was indicative of a neurologically relevant phenotype: following a “startle,” the distance traveled and number of direction changes both significantly increased for treated fish [38].

Here, two groups of gene sets gene-class analyses were conducted: ND (“neurological disorder”) and ASD (“autism spectrum disorder”). The gene sets in the ND collection, known to be associated with a variety of human neurological disorders, are described in Table 2. The gene sets in the ASD collection, associated with enriched gene expression in autism, are described in Table 3. Both gene sets are provided in Supporting Information (Table S1, “ND gene list,” and Table S2, “ASD gene list”). Each group consisted of a collection of gene sets, with each set tested against the ranked list of genes reflecting signal-to-noise ratio of MIX treatment (combining FLX, VNX & CBZ) relative to control. Additional comparisons between the control and treatments consisting of the three pharmaceuticals considered individually were included for comparison (Tables 6, 7).

Gene-class analyses used GSEA release 2.06 and MSigDB release 2.5. Weighted enrichment scores were calculated using gene expression lists ranked by signal-to-noise ratio. The genes on the array were ranked by correlation between the MIX and CTL treatments (those genes with the strongest up-regulation in treatment relative to control were ranked highest; those with strongest down-regulation were ranked lowest). (See Table S3, “Ranked gene list,” for these data.) The maximum gene set size was set to 500 genes; the minimum gene set size was set to 10 genes; the number of permutations was set to 1000. Permutations were conducted by gene set (rather than by phenotype). For details of GSEA parameter usage, see Subramanian et al. [36].

Gene sets were examined to ensure they contained only GSEA-recognized primary HUGO symbols, rather than aliases or unapproved symbols. This was accomplished through the use of a custom script that compared each gene in a given set to the GENE_SYMBOLS.chip file (from GSEA) containing a list of HUGO symbols with accepted aliases. Gene set components listed as aliases in this file were replaced with the appropriate HUGO symbol. For additional details of the annotation and GSEA implementation using the EcoArray 15k Fathead Minnow arrays, see Thomas et al. [37].

## Supporting Information

Table S1 **ND gene list.** A list of the neurological disorders gene sets, in the GSEA gene matrix (.GMX) file format.
(GMX)

Table S2 **ASD gene list.** A list of the autism spectrum disorders gene sets, in the GSEA gene matrix (.GMX) file format.
(GMX)

Table S3 **Ranked gene list.** A Microsoft Excel spreadsheet containing all annotated array gene elements sorted by signal-to-noise ratio for the mixture v. control comparison.
(XLS)
